# Activation of the DNA Damage Response by RNA Viruses

**DOI:** 10.3390/biom6010002

**Published:** 2016-01-06

**Authors:** Ellis L. Ryan, Robert Hollingworth, Roger J. Grand

**Affiliations:** School of Cancer Sciences, College of Medicine and Dentistry, University of Birmingham, Birmingham B15 2TT, UK; exr729@student.bham.ac.uk (E.L.R.); rxh291@student.bham.ac.uk (R.H.)

**Keywords:** RNA viruses, retroviruses, DNA damage response, HIV-1, HTLV-1, HCV, Influenza A, IBV

## Abstract

RNA viruses are a genetically diverse group of pathogens that are responsible for some of the most prevalent and lethal human diseases. Numerous viruses introduce DNA damage and genetic instability in host cells during their lifecycles and some species also manipulate components of the DNA damage response (DDR), a complex and sophisticated series of cellular pathways that have evolved to detect and repair DNA lesions. Activation and manipulation of the DDR by DNA viruses has been extensively studied. It is apparent, however, that many RNA viruses can also induce significant DNA damage, even in cases where viral replication takes place exclusively in the cytoplasm. DNA damage can contribute to the pathogenesis of RNA viruses through the triggering of apoptosis, stimulation of inflammatory immune responses and the introduction of deleterious mutations that can increase the risk of tumorigenesis. In addition, activation of DDR pathways can contribute positively to replication of viral RNA genomes. Elucidation of the interactions between RNA viruses and the DDR has provided important insights into modulation of host cell functions by these pathogens. This review summarises the current literature regarding activation and manipulation of the DDR by several medically important RNA viruses.

## 1. RNA Viruses

Viruses that use RNA as their genetic material are a diverse group of obligate parasites associated with some of the most infectious and deadly human diseases. The genomes of RNA viruses are highly varied and can be either double-stranded or single-stranded, with the latter being of negative or positive polarity. RNA virus genomes can also be either segmented, whereby each segment typically encodes one viral protein, or unimolecular, in which a single polyprotein is translated and cleaved to form individual viral proteins [1]. The genetic diversity of RNA viruses stems from a high rate of spontaneous mutation and has made development of antiviral therapies and vaccines extremely challenging [2]. The increased mutation frequency is primarily due to a lack of proofreading by the RNA-dependent RNA polymerases (RdRp) required for replication of many RNA viruses [3]. This elevated mutation rate is believed to be the reason that RNA virus genomes are usually restricted to lengths of less than 30 Kb and therefore tend to be smaller and encode fewer proteins than those of many DNA viruses [4].

RNA viruses have diverse modes of replication that largely depend on their genome configuration. In positive-sense single-stranded RNA viruses, such as coronaviruses and hepatitis C virus (HCV), the viral genome functions as mRNA and can be directly translated into a viral polyprotein by a host ribosome. Viral proteins can then form membrane-associated replication complexes (RC) in the cytoplasm that provide sites for viral RNA synthesis [5]. Negative-strand RNA viruses, such as Rabies and Influenza, must initially use a viral RdRp to produce monocistronic mRNAs. These mRNAs are translated to produce viral proteins while production of new viral genomes first requires synthesis of positive-sense RNA which is then converted to negative-sense viral genomes. In this case, virus replication and assembly requires formation of ribonucleoprotein (RNP) complexes that contain viral polymerases and nucleoproteins [6].

Retroviruses are a unique class of RNA viruses whose lifecycles contain a DNA intermediate that requires integration into the host cell genome prior to viral gene expression. The retrovirus genome consists of two identical positive-strand RNA molecules of between 7 and 12 kb that are capped at the 5' end and polyadenylated at the 3' end [7]. Following cell entry, viral reverse transcriptase (RT) enzymes are used to generate double-stranded DNA (dsDNA) molecules from the RNA templates. These are then transported to the nucleus and integrated into cellular DNA which requires another virus-encoded enzyme known as integrase (IN) [8]. The viral mRNA is exported to the cytoplasm where it is translated to viral proteins and the remainder is packaged as new viral genomes. Since integration of the viral cDNA requires breaks in cellular DNA, activation of host DNA repair pathways is a consequence of retroviral infection that is discussed in detail below.

For some RNA viruses, such as members of the families *Orthomyxoviridae* and *Retroviridae*, at least part of the replication cycle takes place in the nucleus. For the majority, however, replication occurs exclusively in the cytoplasm and therefore the impact of the viral lifecycle on the nucleus may be less severe than is the case for many DNA viruses. However, proteins encoded by RNA viruses are often transported to the nucleus where they can perturb cellular functions and inhibit the antiviral response [9]. As is discussed below, these nuclear activities may involve the direct or indirect introduction of DNA damage and impairment of the subsequent cellular response.

## 2. The Cellular DNA Damage Response and DNA Repair Pathways

The integrity of the genome is under constant attack from agents able to damage DNA. DNA damage can be generated from both endogenous cellular processes and exogenous agents, and it has been estimated that cells accumulate tens of thousands of lesions per cell per day [10]. Endogenous cellular processes that cause DNA damage include the generation of reactive oxygen species (ROS) that generate base damage, DNA polymerase errors during replication that can cause DNA mismatches and the abortive action of enzymes, such as DNA topoisomerase I (TOPI) and DNA topoisomerase II (TOPII) that can lead to DNA strand breaks [11]. Exogenous agents such as ultra-violet (UV) radiation and ionising radiation (IR), as well as infection with a range of microorganisms, can also result in significant DNA damage [12].

**Figure 1:**
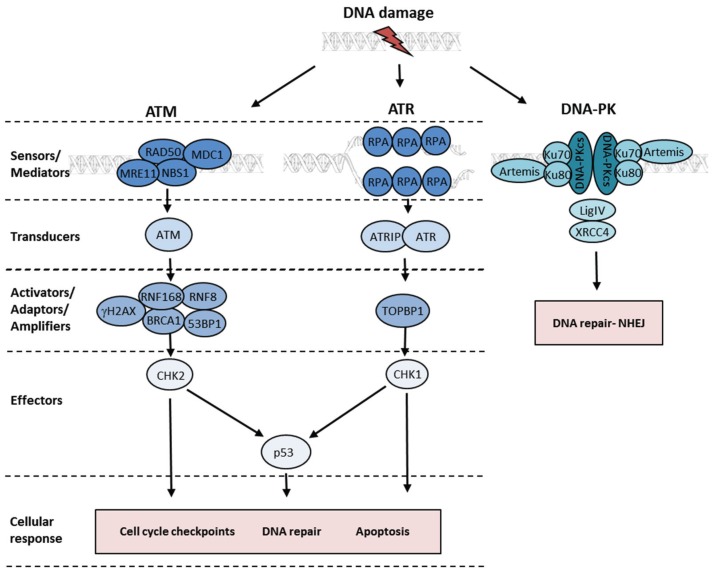
ATM, ATR and DNA-PK signalling pathways. The DNA damage signalling pathways are primarily mediated by the ATM, ATR and DNA-PK kinases. DSBs are detected by the MRN complex (MRE11, RAD50 and NBS1) which recruits and activates ATM at the site of the break. Activated ATM phosphorylates numerous effector proteins such as CHK2 and p53. Phosphorylation of the histone variant H2AX by ATM and ubiquitination of H2AX by RNF8 and RNF168 E3 ubiquitin ligases precedes recruitment of repair proteins such as BRCA1 and 53BP1. ATR is primarily activated at regions of ssDNA which are first coated by RPA. ATR is then recruited to RPA-coated ssDNA via its interacting protein ATRIP. The recruitment of TOPBP1 to the ssDNA region is responsible for the activation of ATR which subsequently phosphorylates effector proteins such as CHK1 and p53. In contrast to the ATM and ATR kinases, DNA-PK is responsible for the regulation of the NHEJ DSB repair pathway. Image adapted from [15].

The rapid and accurate repair of DNA lesions is vital for cellular survival and for the maintenance of genome stability. If not accurately repaired, DNA damage can cause erroneous changes in the genetic code leading to increased mutational load and an elevated risk of developing cancer [10]. To respond to genotoxic lesions induced by DNA damaging agents, cells have evolved complex mechanisms for DNA damage detection and repair, collectively termed the DNA damage response (DDR). The DDR pathways comprise a highly coordinated network of proteins which are rapidly activated in the presence of DNA damage. The DDR is composed of sensors, transducers and effectors, which together form a signalling cascade involving complex interactions and post-translational modifications (Figure 1). Initiation of this cascade leads to cell cycle arrest and the activation of DNA repair pathways. The DDR is principally mediated by the PI3K kinase family: ataxia telangiectasia mutated (ATM), ATM and Rad3-related (ATR) and DNA-dependent protein kinase (DNA-PK), and by members of the poly (ADP-ribose) polymerase (PARP) family. ATM and DNA-PK are primarily activated by double-strand breaks (DSBs); whilst ATR is stimulated at regions of single-stranded DNA (ssDNA) generated at DSBs or stalled replication forks [13]. PARP1 is primarily involved in the detection and repair of single-strand breaks (SSBs) [14].

## 3. The ATM Kinase and the Detection of Double-Strand Breaks

DSBs are discontinuities in both strands of the DNA double-helix and it is thought that a single cell can accumulate up to 10 endogenously-induced DSBs per day [16]. An early event following DSB formation is the detection of the break by the DSB sensor complex, MRE11-RAD50-NBS1 (MRN). The MRN complex recruits and activates the transducer kinase ATM at the site of the break [17,18]. Active ATM is then able to phosphorylate downstream effector proteins of the DDR including histone H2AX as well as cell cycle checkpoint proteins p53 and CHK2, which promote cell cycle arrest to prevent replication of damaged DNA [19]. Phosphorylated H2AX, referred to as γH2AX, allows binding of the mediator of DNA damage checkpoint 1 (MDC1) at the break, where it is subsequently phosphorylated by ATM [20]. Ubiquitination of histones H2A and H2AX by the E3 ubiquitin ligases RNF8 and RNF168 precedes the recruitment of a range of DNA repair proteins including breast cancer type 1 susceptibility protein (BRCA1) and p53-binding protein 1 (53BP1) [21,22].

## 4. ATR Kinase Activation at Single-Stranded DNA

ATR is activated in response to numerous types of DNA damage that result in the formation of ssDNA [23]. Regions of ssDNA are rapidly coated by replication protein A (RPA), which recruits the transducer protein ATR to the site of the lesion via its interaction with ATR-interacting protein (ATRIP) [23]. A series of protein complexes (the Rad17-replication factor C 2 (RFC2) clamp loader complex and the Rad9-Rad1-Hus1 (9-1-1 complex) are consecutively recruited to the ssDNA-RPA complex, ultimately resulting in the localisation of topoisomerase II binding protein 1 (TOPBP1). TOPBP1 is able to activate ATR via interactions with ATRIP and Rad9 of the 9-1-1 complex [24]. This leads to the phosphorylation and subsequent activation of downstream effector proteins, such as p53 and CHK1, which induce cell cycle arrest and DNA repair [19].

## 5. The DDR and Cell Cycle Checkpoints

One of the primary functions of the DDR is to initiate cell cycle arrest following detection of DNA damage, thus preventing the replication of mutated DNA [25]. Three major cell cycle checkpoints have been defined: the G1/S checkpoint is activated to stop the initiation of replication in the event of DNA damage, the intra S-phase checkpoint can be activated in response to replication stress and slows DNA synthesis, and the G2/M checkpoint which ensures that damaged chromosomes are not segregated to the daughter cells during mitosis. Activation of these checkpoints following DNA damage depends on the activities of the kinases ATM, ATR, CHK1 and CHK2.

Activated CHK1 and CHK2 can rapidly activate the G1/S checkpoint by phosphorylating and inactivating the phosphatase Cdc25A which prevents the activation of the Cyclin E-Cdk2 complex thereby inhibiting S-phase entry [26]. CHK1 and CHK2 can also maintain G1/S arrest through phosphorylation and stabilisation of p53. Activated p53 can increase the expression of p21 which inhibits Cyclin E-Cdk2 activity [27]. The intra S-phase checkpoint can also be activated through phosphorylation of Cdc25A resulting in the prevention of Cdc45 loading and firing of replication origins [28]. In addition, ATM can also signal through an alternative pathway during S-phase requiring the MRN complex and phosphorylation of structure maintenance of chromosome 1 (SMC1) [29]. CHK1 and CHK2 can initiate G2/M arrest by inhibiting activation of the cyclin B-Cdk1 complex via phosphorylation and degradation of Cdc25C [30]. The stabilisation of Wee1 following DNA damage acts to maintain G2 arrest through increased phosphorylation of Cdk1 [31].

## 6. Double-Strand Break Repair

The two main DSB repair mechanisms are non-homologous end joining (NHEJ) and homologous recombination (HR) repair. NHEJ involves a coordinated network of proteins which facilitate rapid repair during all stages of the cell cycle. During NHEJ, the DSB is first recognised by the Ku70/80 heterodimer, which forms a ring-like structure around either end of the DSB. Association of Ku70/80 leads to recruitment of the catalytic subunit of the DNA-PK (DNA-PKcs) to form the DNA-PK holoenzyme [32]. Before DNA ends can be re-ligated, they must be restored to their conventional 3'OH and 5'P termini by end-processing enzymes such as Artemis. DNA ligase IV, in complex with XRCC4 and XLF, performs DNA end-joining of the DSB termini, completing NHEJ [33,34,35]. Occasionally, NHEJ can result in the loss of nucleotides leading to inaccurate repair. In addition, DNA ends from other regions of the genome can be erroneously re-ligated, resulting in rearrangements and chromosomal translocations [36].

In contrast to NHEJ, HR occurs exclusively during S and G2 phases of the cell cycle and uses the undamaged homologous sister chromatid as a template for accurate repair. The DSB is first processed by endonucleases to produce a 3' overhang which is coated by RPA. RPA is then displaced by the recombinase RAD51 to form a nucleoprotein filament that invades the undamaged strand of the homologous sister chromatid forming a displacement loop (D-loop) [37]. The nucleoprotein filament searches for the undamaged sister chromatid which is used as a template for filling in the missing information on the damaged strand. HR can result in branched structures known as Holliday junctions that must be resolved by endonucleases to restore the two separate strands [38].

## 7. Repair of Single-Strand DNA Damage

Most commonly occurring DNA lesions affect only a single DNA strand and include SSBs, modifications to individual nitrogenous bases and replication errors that introduce mismatched nucleotides [39]. Mechanisms that correct single-strand damage can utilise the undamaged DNA strand as a template to ensure accurate repair. Although SSBs are less genotoxic than DSBs, their rapid repair is critical to the maintenance of genome integrity since they can collapse into DSBs if met by a replication fork during DNA replication. Multiple DNA repair pathways have been identified that correct single-strand damage.

Base excision repair (BER) corrects damage to single mismatched or modified bases and is the principle pathway used for the reversal of oxidative DNA damage [40]. During BER, base damage is detected by DNA glycosylases that excise the damaged base creating an apurinic-apyrimidinic (AP) site [41]. The AP endonuclease 1 (APE1) incises the AP site, generating a single-stranded nick [42]. DNA polymerases fill the damaged region which is then sealed by either DNA ligase I or ligase IV [43].

Nucleotide excision repair (NER) is responsible for the removal of helix-distorting, bulky lesions, such as cyclobutane pyrimidine dimers (CPDs) and (6-4) photoproducts, that can be induced by UV radiation [44]. In NER, recognition of the damaged region is followed by excision of the bulky lesion by ERCC1-XPF and XPG endonucleases [45]. Gap filling is carried out by the DNA polymerases Pol δ and Pol ε in conjunction with PCNA and ligation by the ligase III-XRCC1 complex [46,47].

DNA base mismatching that occurs due to DNA polymerase errors can be repaired by members of the mismatch repair (MMR) pathway which acts exclusively on the newly synthesised DNA strand. Following detection of mismatched bases by MSH2-MSH6 (MutSα) or MSH2-MSH3 (MutSβ) complexes, the MLH1/PMS2 (MutLα) complex is then recruited followed by the exonuclease EXO1 which resects the damaged strand [48,49,50,51]. The DNA polymerase Pol δ fills the gap in an RFC and PCNA-dependent manner while DNA ligase I ligates the newly synthesised strand [52,53].

## 8. DNA Damage and Apoptosis

Extensive DNA damage that cannot be repaired can lead to activation of apoptotic pathways and this response represents a crucial barrier against malignant transformation [54]. Increased p53 transcriptional activity following phosphorylation by DDR kinases induces expression of pro-apoptotic proteins such as Bax, Noxa and Puma that trigger cell death via regulation of mitochondrial permeability [55]. Apoptotic pathways can also be activated in response to RNA virus infection as part of the innate and adaptive immune responses to invading pathogens, although some viruses take advantage of apoptotic machinery to aid viral maturation and dissemination. Regardless of the impact on the viral lifecycle, apoptosis can contribute significantly to viral pathogenesis. For example, the human immunodeficiency virus 1 (HIV-1) Vpr protein can promote apoptosis by targeting the mitochondrial permeability transition pore complex and this may contribute to depletion of CD4+ lymphocytes *in vivo* [56]. In addition, apoptosis induced by the Influenza A virus NS1 protein contributes to lung tissue destruction and widespread cell death and may cause further damage through aberrant inflammatory responses [57].

Since apoptosis occurring prior to production of infectious progeny will have a detrimental effect on viral propagation, RNA viruses have also evolved mechanisms to inhibit or delay the process. The HCV NS3-4A protease contributes to viral immune evasion by cleaving the TRIF component of the Toll-like receptor signalling pathway that could otherwise trigger cell death following recognition of viral RNA [58]. HCV core protein can also promote cellular survival by activation the NF-κB pathway and this could contribute to the oncogenic potential of the virus [59]. In viruses that induce DNA damage early in their lifecycles, impairment of downstream signalling to apoptotic machinery is often necessary to ensure successful completion of viral replication.

## 9. Non-Coding RNAs and the DDR

It is now apparent that a number of small non-coding RNA (ncRNAs) molecules have important roles in regulation of the DDR. These include microRNAs (miRNAs) that can inhibit gene expression by binding to, and promoting degradation of, messenger RNAs (mRNAs). The miR-34 family of miRNAs, for example, are transcriptional targets of p53 following DDR activation and create a positive feedback loop by repressing expression of HDM4, a negative regulator of p53 [60]. The importance of miRNAs in the DDR is also highlighted by the fact that depletion of miRNA processing proteins such as DICER and AGO2 inhibits repair of damaged DNA [61]. An additional set of RNAs that have been reported to play a significant role in the DDR are known as DROSHA- and DICER-dependent small RNAs (DDRNAs) [62,63]. They are transcribed from DNA sequences close to the site of damage and have important roles in the formation of the protein aggregations known as DNA damage foci. A variety of possible roles for the DDRNAs have been proposed, including acting as templates for DNA polymerase, as guides for the recruitment of repair proteins or as scaffolds for the repair foci themselves [64]. Long non-coding RNAs (LncRNAs), RNAs of more than 200 nucleotides in length, are also expressed following DNA damage. It has recently been shown that the lncRNA, DDSR1, regulates levels of BRCA1 and RAP80 at sites of DSBs as well as interacting with BRCA1 and hnRNPUL1, an RNA binding protein with a role in DNA end resection [65].

## 10. Viral Interaction with the DDR

Many viruses are known to cause activation of the DDR during their lifecycles. This may occur as an indirect consequence of cellular stress caused by viral infection or as part of an antiviral response that aims to deactivate the invading viral genomes. In addition, viral DNA may be recognised as damaged cellular DNA while many viral proteins have been shown to cause DNA lesions directly. The DDR may have either beneficial or detrimental effects for the virus, enhancing or inhibiting viral replication. When activation of the DDR is deleterious, viruses may deactivate particular pathways while still making use of certain other elements. For example, adenovirus genomes are joined end to end to form concatemers by the host cell DDR following infection with various disabled viruses [66]. However, the wild type virus is able to hi-jack cellular ubiquitin E3 ligases to target significant DDR proteins for rapid proteasome-mediated degradation [67]. Despite this, several DDR factors are recruited to adenovirus replication centres where they are involved in viral DNA synthesis [68,69,70]. Other viruses, whilst activating the DDR, make more obvious use of its components. For example, optimal production of progeny simian virus 40 (SV40) requires phosphorylation of large T antigen by the ATM kinase [71,72]. On the other hand, SV40 also initiates degradation of MRE11, a protein involved in detecting DSBs [73,74]. The larger DNA viruses, such as members of the *Gammaherpesvirinae* family, have a similarly intricate relationship with the DDR. Epstein-Barr virus (EBV), for example, induces genome instability and DDR activation through the action of a number of viral proteins including EBNA-1 and LMP-1 [75,76,77,78]. However, other EBV proteins, such as EBNA3C, can attenuate downstream DDR signalling that could have a negative impact on the viral lifecycle [79,80].

The complex relationship between DNA viruses and the DDR has been summarised in several recent literature reviews [15,81,82]. Other published reviews have focused on specific aspects of these interactions, such as the effect on the cell cycle and ubiquitination, [83,84] or on particular viruses in isolation [85,86,87]. The literature relevant to RNA viruses and the DDR is much more limited and has so far focused on HCV and the retroviruses HIV-1 and human T-cell lymphotropic virus 1 (HTLV-1) [88,89,90,91].

## 11. RNA Viruses and DNA Damage

The following text will examine the introduction of DNA damage following infection with RNA viruses as well as considering the subsequent activation and modulation of host DDR pathways. Examples include those from both true RNA viruses, which mostly replicate in the cytoplasm, and retroviruses, whose lifecycle includes a DNA intermediate that integrates into the host cell genome. A summary of interactions between several RNA viruses and the DDR is also presented in Table 1.

**Table 1:** Overview of several RNA viruses which impact on the cellular DDR.

Virus	Family	RNA Genome Conformation	DDR Consequences	Representative References
Human immunodeficiency Virus 1 (HIV-1)	*Retroviridae*	+ single strand (2 copies)	Activation of ATR, replication stress, activation of nucleases and formation of DDR foci by Vpr	[92,93,94,95]
Human T-cell lymphotropic virus 1 (HTLV-1)	*Retroviridae*	+ single strand (2 copies)	Genome instability and DNA damage; attenuation of BER, NER, MMR, HR, NHEJ pathways by Tax. Generation of ROS	[96,97,98]
Hepatitis C virus (HCV)	*Flaviviridae*	+ single strand	Generation of ROS and NO, reduced expression of MMR, BER and NER components, interaction of viral proteins with ATM and modulation of ATM pathway activity	[99,100,101,102,103]
Infectious bronchitis virus (IBV)	*Coronaviridae*	+ single strand	Activation of ATR pathway and DNA replication stress	[104]
Influenza A virus	*Orthomyxoviridae*	− single strand	Direct DNA damage (comet assay) Induction of γH2AX foci	[105,106]
Chikungunya virus	*Togaviridae*	+ single strand	Induction of GADD34 expression	[107]
Sindbis virus	*Togaviridae*	+ single strand	Activation of PARP-1	[108]
La Crosse virus	*Bunyaviridae*	− single strand	Increased phosphorylation of H2AX	[109]
Rift valley fever virus (RVFV)	*Bunyaviridae*	− single strand	Activation of ATM signalling; inhibition of ATR	[110]
Avian Reovirus (ARV)	*Reoviridae*	double strand	Genome instability and activation of ATR signalling	[111]

## 12. Retroviral Integration and the DDR

**Figure 2:**
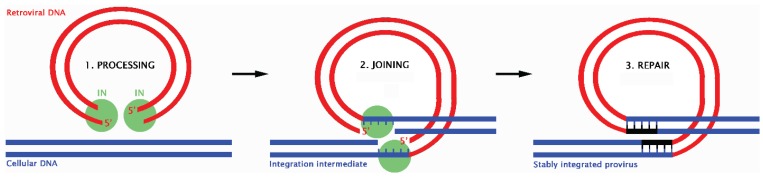
The basic steps in the retroviral integration process. 1. Viral integrase (IN) processes the viral DNA ends. 2. IN then catalyses the cleavage of host DNA and attachment of viral DNA forming an integration intermediate. 3. Finally, host factors repair the single strand gaps resulting in a stably integrated provirus. Image adapted from [120]. (2003) National Academy of Sciences, USA.

Retroviruses are a unique class of intracellular parasites that include HIV-1 and HTLV-1. In order to replicate, retroviruses must reverse transcribe their RNA genome and integrate the subsequent DNA intermediate into the chromosomes of host cells (Figure 2). The viral integrase protein is essential in this process as it first catalyses the removal of two nucleotides from the 3'ends of the viral DNA and then participates in the joining of the exposed hydroxyl groups to staggered phosphates in the host chromatin [90]. Insertion of retroviral DNA results in the creation of DSBs and leaves single-strand gaps that flank the integration site and link viral and host DNA [90]. Efficient repair of these lesions is vital for host cell integrity and subsequent transcription of the viral genome. Since retroviral proteins with the ability to repair this damage have not been identified, it has long been speculated that host enzymes must fulfil the role [112].

Several studies examining the involvement of the host DDR in retroviral replication have identified the NHEJ pathway as central to post-integration DNA repair [113,114,115,116,117,118]. The infection of murine cells lacking DNA-PK, an essential enzyme in NHEJ, with three different retroviruses resulted in reduced viral DNA integration and high levels of apoptosis [113]. In addition, depletion of Ku80, another important NHEJ factor, has been shown to impede HIV-1 replication significantly [115,118]. The NHEJ pathway has also been implicated in the circularisation of unintegrated viral DNA that would otherwise be sensed as DNA damage by the cell and lead to apoptosis [114,115]. While there is significant evidence for the involvement of NHEJ in retroviral post-integration repair, efficient lentivirus transduction has been demonstrated in DNA-PK deficient cells suggesting that, at least in some circumstances, alternative repair pathways could be involved [119].

An examination of the role of the ATM and ATR kinases in retroviral integration has resulted in several conflicting reports. A reduction in retroviral transduction was observed in cells transfected with a dominant-negative form of the ATR gene but not in ATM-deficient cells, suggesting that the retroviral lifecycle relies on an ATR-dependent DDR [120,121]. In contrast, another study found that a small molecule inhibitor of ATM was able to suppress replication of both wild type and drug-resistant HIV-1, indicating a key role for this kinase in the viral lifecycle [122]. To add to the confusion, other researchers have questioned the importance of either kinase by showing that RNA interference of ATM and ATR as well as inhibition with caffeine (a general kinase inhibitor) had no effect on the efficiency of HIV-1 transduction or retroviral-induced apoptosis [123,124]. Subsequently, it was shown that the involvement of these DDR kinases in retroviral replication can vary between cell types which may partly explain the discrepancies observed in earlier studies [112].

PARP-1 has been implicated in SSB and DSB repair, DNA replication, genome stability, transcription and apoptosis [125]. As with ATM and ATR, there has been considerable disagreement over the role of PARP-1 in retroviral integration. Following experimental suppression of PARP-1 activity, several studies have claimed that this enzyme is essential for HIV integration [126,127,128] while others have stated that it is dispensable [119,123,129]. As well as the use of varying experimental conditions, the fact that PARP-1 is known to have overlapping roles with other members of the ARTD(PARP) family in mammalian cells may explain the lack of consensus in these reports [130,131]. More recently it has been shown that, rather than effecting viral DNA integration, PARP-1 promotes transcriptional repression of viral DNA post-integration via epigenetic mechanisms [132].

It is believed that minor SSBs can be repaired independently of ATM and ATR using the BER pathway [133]. A targeted siRNA screen against DNA repair genes revealed that inhibition of several factors involved in the short-patch BER pathway impeded HIV-1 replication [134]. Surprisingly, genes involved in NHEJ did not feature prominently in the screen [134]. A subsequent report indicated that the BER pathway is important for post-integration repair during lentiviral infection but less so for other retroviruses [135]. It was suggested that the sequence site preference for integration unique to each retrovirus may determine the choice of pathway for the subsequent repair process [135].

Questions remain over the importance of host DDR proteins in retroviral replication. The conflicting results from these studies may stem from the use of different cell types, many of which are not necessarily a representative model of *in vivo* retroviral replication, combined with the use of distinct vector-based transduction methods. In addition, many conclusions have been drawn from the use of non-specific inhibitors that are known to have off-target effects while negative RNA interference data may simply indicate that expression of only a small amount of a given protein is required during the replication process [112]. There is also evidence that the process of post-integration repair may be separate from classic DNA repair pathways which raises the possibility of inhibiting this process while leaving important cellular functions intact [136]. This could be particularly significant given that targeting of virally encoded proteins to treat HIV-1 infection has proved problematic due to the high mutation rate and short replication cycle of the virus [122].

## 13. Human Immunodeficiency Virus 1 (HIV-1) Vpr Protein

HIV-1 is a lentivirus that belongs to the *Retroviridae* family. Lentiviruses are characterised by an extended incubation period and are associated with chronic diseases that affect the immune and central nervous systems [137]. HIV-1 is well known as the causative agent of acquired immune deficiency syndrome (AIDS) which develops via the progressive depletion of CD4+ T cells. Along with structural and regulatory proteins, HIV-1 also encodes several accessory proteins which can contribute to HIV infectivity and pathogenesis [93]. The 14 kDa viral protein R (Vpr) is one such accessory molecule that has been studied extensively in the context of its interaction with the host DDR. Vpr has multiple functions in the HIV-1 infection process including viral and host gene transcriptional co-activation, nuclear import of viral DNA, modulation of NF-κB activity as well as cell cycle and apoptotic regulation [138].

Vpr is able to modulate the host cell cycle and can arrest lymphocytes at G2/M by inhibiting activation of the cyclin B-Cdk1 complex [139,140,141,142]. Following cell cycle arrest, Vpr expression can promote apoptosis in infected cells [143,144,145,146] and this may contribute to the *in vivo* depletion of CD4+ T lymphocytes [138]. Regulation of the host cell cycle is believed to offer significant advantages to the virus during the early stages of infection [147]. There is also evidence that viral genome expression is optimal in G2 phase and that Vpr arrests cells at the point where the HIV-1 long terminal repeat (LTR) is most active in order to increase transcription of viral genes [148]. In addition, the presence of an internal ribosome entry site (IRES) in the HIV-1 genome could selectively enhance the translation of viral proteins during G2 arrest [149].

Expression of Vpr can cause DNA damage and activate DDR pathways and this has been proposed as the mechanism by which Vpr induces cell cycle arrest in G2. Using a pulsed-field gel electrophoresis (PFGE) assay, it was shown that Vpr can induce DSBs and that formation of these DNA lesions was dependent on the ability of Vpr to bind to chromatin [94]. The same group subsequently showed that Vpr expression activates the ATM-CHK2 pathway and upregulates HR repair which was demonstrated by an increase in formation of RAD51 and BRCA1 foci [150]. The authors suggest that DSB formation and ATM activation by Vpr may enhance HIV-1 integration efficiency as well as contributing to HIV-1 pathogenesis.

Several studies have shown that activation of the ATR-CHK1 pathways is specifically required for Vpr-mediated G2 arrest [93,151,152,153]. One study demonstrated that Vpr can bind directly to host DNA, potentially interfering with DNA replication and resulting in the accumulation of chromatin-associated RPA which then activates ATR [93]. The same study failed to detect DSB formation or ATM activation following Vpr expression suggesting that the ATM-CHK2 arm of the DDR may be dispensable for Vpr-induced cell cycle arrest. Additional studies have provided evidence that Vpr causes replication stress in CD4+ T cells that leads to formation of RPA foci and activation of ATR and several downstream substrates [95,153]. However, it has subsequently been argued that Vpr induces G2 arrest via a molecular mechanism that is distinct from that induced by DNA damage or replication stress [154,155]. It was shown that exposing cells to agents that typically induce ATR/CHK1 activation, such as UV and hydroxyurea, resulted in Cdc25A degradation and S-phase arrest. Although expression of Vpr also caused phosphorylation of CHK1 in S-phase, the Vpr-expressing cells were able to proceed to G2 with Cdc25A levels largely unaffected [155]. These finding are supported by an earlier study using inhibitors of DDR kinases which found that accumulation of cells in G2 is mediated by Vpr in a mechanism that is distinct from that induced by DNA damage [144].

Vpr-induced cell cycle arrest via ATR activation has also been linked to the manipulation of the ubiquitin proteasome system (UPS). Manipulation of the host UPS has been observed in several unrelated viruses including human papillomavirus (HPV) [156], hepatitis B virus (HBV) [157] and paramyxovirus (PMV) [158] and can induce the polyubiquitination and degradation of cellular factors that ultimately aid viral replication. Vpr binding of both Cul4A and DDB1, components of the DDB1-CUL4A-RBX1 E3 ligase complex, via interaction with VPRBP is believed to cause ATR activation and G2 arrest through the ubiquitination of an unknown target [159,160]. Inhibition of K48 polyubiquitination significantly reduces the formation of Vpr-induced γH2AX foci but not UV-induced γH2AX foci [161]. It has been postulated that Vpr may trigger degradation of DNA repair regulators and lead to recruitment of DNA repair proteins in the absence of replication stress [161]. More recently, Vpr was found to interact with SLX4, a nuclease scaffold protein involved in the Fanconi anemia repair pathway [92]. Recruitment of VPRBP and PLK1 following Vpr-SLX4 binding was shown to cause premature activation of SLX4-associated MUS81-EME1 endonucleases leading to replication stress through abnormal processing of stalled replication forks. The Vpr-SLX4 interaction led to an accumulation of FANCD2 foci, indicating DNA damage in S-phase, and was also required for Vpr-mediated G2/M arrest. The presence of Vpr and SLX4 also suppressed induction of type 1 interferon by HIV-1 and it was suggested that Vpr may induce SLX4 to process viral DNA that would otherwise accumulate and trigger an immune response.

The ability of Vpr to cause cell cycle arrest in G2 is a well-established phenomenon although the mechanism behind it is still under debate. Vpr expression can increase the occurrence of several markers of DNA damage and, in addition to the studies mentioned above, Vpr has also been linked to an increase in intracellular ROS levels which are known to cause DNA damage during infection by several RNA viruses [162,163]. Despite these observations, the requirement for DDR activation in cell cycle modulation by Vpr has been questioned by more than one study suggesting that downstream signalling may be modulated by HIV-1 [144,155]. The capacity of Vpr to induce cell cycle arrest and apoptosis in a p53-independent manner has led to the proposal that it could function as an anti-cancer agent if delivered via a genetically modified oncolytic virus [164]. However, as with other chemotherapeutic agents, its effectiveness may be limited by the potential of Vpr to induce significant genetic instability in otherwise healthy cells [94,165].

## 14. Human T-Cell Lymphotropic Virus Type 1 (HTLV-1) Tax Protein

HTLV-1 is another member of the *Retroviridae* family and the etiological agent of the aggressive CD4+ T cell proliferative malignancy, adult T-cell leukemia (ATL), as well as a progressive neurological disease known as HTLV-1-Associated Myelopathy/Tropical Spastic Paraparesis (HAM/TSP). As well as the structural proteins common to all retroviruses, HTLV-1 also encodes a number of regulatory and accessory proteins that include a 40 kDa viral transactivator known as Tax. Tax has been classified as a viral oncoprotein and is essential for cellular transformation following HTLV-1 infection [166]. As well as activating cellular pathways that promote cellular proliferation and interfering with tumour suppressor function, Tax can also disrupt cell cycle progression, introduce DNA damage and interfere with DNA repair [167].

Tax expression can increase intracellular ROS levels through an interaction with ubiquitin-specific protease 10 (USP10), which inhibits its antioxidant activity, as well as through activation of the transcription factors NF-κB and CREB [168,169]. ROS induced by Tax has been shown to cause DNA damage in T cells and fibroblasts and increase the expression of the senescence marker SEN1 [170]. The ability of Tax to activate the NF-κB pathway has also been linked to the formation of DSBs due to stimulation of nitric oxide (NO) production [171]. As well as increasing ROS/NO-induced DNA damage, Tax expression also leads to down-regulation of DNA polymerase β, a key component of the BER pathway that is primarily responsible for correcting oxidative DNA lesions [172]. An additional study found BER repair was impaired in HTLV-1-infected cells and in those expressing Tax alone [96].

HTLV-1 is known to enhance proliferation of infected cells and multiple studies have implicated Tax in dysregulation of the cell cycle [89]. Tax can both impair the recruitment of MDC1 to repair foci and reduce phosphorylation of H2AX and RPA by enhancing expression of the cellular phosphatase WIP1 [98,173]. Inference with activation and recruitment of key DDR proteins allows Tax-expressing cells to enter S-phase with unrepaired DNA damage. Tax expression can also accelerate S-phase progression through interaction with the MCM2-7 complex and replication origins [174]. The unlicensed firing of replication origins can result in replicative stress and genomic lesions. In line with these findings, a separate study showed that ectopic expression of Tax leads to DSB formation during DNA replication in S-phase [97]. In addition, the authors demonstrated that HR repair of these lesions was compromised in favour of the more error-prone NHEJ repair pathway. A subsequent study by the same group confirmed that Tax can interfere with host DNA replication leading to firing of backup replication origins and DNA damage [171]. Increased DNA damage following expression of Tax in cells already deficient in HR provides evidence that Tax can cause damage independently of its ability to depress HR function.

Tax can also interact with cell cycle checkpoint kinases and compromise the ability of the host cell to arrest and engage DNA repair pathways following DNA damage. Tax can bind to CHK1, inhibit its kinase activity and attenuate the G2 checkpoint following treatment with IR [175]. Tax can also interact with the kinase domain of CHK2 and impair its role in the DDR by inhibiting its dissociation from chromatin [176]. Tax and CHK2 co-localise in nuclear speckled structures that also contain other DDR factors such as 53BP1 and DNA-PKcs [177,178]. Tax can also abrogate the G1 checkpoint following UV-induced DNA damage in both p53+ and p53− cells [177].

HTLV-1 Tax causes DNA damage in host cells through elevated oxidative stress and stimulation of unregulated DNA replication while concurrently undermining cell cycle checkpoints and multiple DNA repair pathways. While there are many aspects to the oncogenic activities of the Tax protein, it is clear that its ability to introduce DNA lesions and modulate the ensuing cellular response will contribute significantly to the chromosomal instability that characterises HTLV-1-associated malignancies.

## 15. Hepatitis C Virus (HCV)

HCV is a small positive-sense single-stranded RNA virus with a genome of 9.6kb [179]. HCV is hepatotrophic and causes acute and chronic hepatitis, while persistent infection can lead to the development of liver cirrhosis and hepatocellular carcinoma (HCC) [180]. The HCV genome encodes a polyprotein precursor that is cleaved by host and viral proteases to produce at least 10 mature structural and non-structural (NS) proteins [179]. HCV infection can stimulate the production of NO via activation of inducible NO synthase (iNOS) as well as amplifying the release of ROS in host cells [99,100]. The accumulation of DSBs and genetic abnormalities in HCV-infected cells has been attributed to the elevated levels of NO and ROS which can overwhelm host repair mechanisms and potentially drive the progression of HCV-associated malignancies [99,100].

As well as inducing DNA damage, HCV can inhibit host DNA repair pathways, often through the direct interaction of viral proteins with host DDR factors. Human liver hepatocellular carcinoma cells expressing the HCV core protein are less able to repair UV-induced DNA damage than those expressing an empty vector [181]. In addition, reduced expression of the MMR genes MSH2, MLH1, MSH6 and PMS2 has also been reported in multiple cases of HCV-associated hepatocellular carcinoma [182]. BER, and to a lesser extent NER, are responsible for the reversal of oxidative damage to DNA [183]. Reduced expression of the NEIL1 glycosylase, involved in cleaving damaged bases during the initial steps of BER, has been observed in HCV-infected cell cultures and in primary tissue samples from patients with advanced liver disease associated with HCV infection [184]. Furthermore, mice hepatocytes expressing the entire HCV open reading frame (ORF) exhibited defective cell cycle arrest and reduced NER through the down-regulation of Gadd45β [185].

HCV proteins have also been found to interfere with the ATM-CHK2 pathway that is typically activated in response to DSBs. The HCV protease NS3/4A can interact with ATM causing its cytoplasmic translocation and rendering cells more sensitive to DNA damage induced by IR [103]. Furthermore, HCV core protein can interact with NBS1 and inhibit ATM activation by interfering with the formation of the MRN complex at the sites of DSBs [101]. Expression of NS2 alone can lead to phosphorylation of the ATM substrate CHK2, although this viral protein has also been shown to inhibit downstream signalling by mislocalising p53 from the nucleus to the cytoplasm [186]. Despite the observation that HCV proteins can impair ATM signalling, it has also been demonstrated that inhibition of this DDR pathway suppresses replication of HCV. This suggests that HCV may hijack certain DDR factors associated with the ATM pathway in order to replicate its genome [102]. As has been suggested with HIV-1, the use of small molecule inhibitors against ATM could be used to impede viral replication and aid in the treatment of HCV-related diseases [102].

## 16. Infectious Bronchitis Virus (IBV)

IBV is a highly infectious avian gamma-coronavirus that primarily targets cells of the respiratory tract [187]. Coronaviruses comprise a diverse group of enveloped positive-strand RNA viruses that are responsible for several human diseases, most notably the severe acute respiratory syndrome (SARS) epidemic in 2003. Coronaviruses form replication complexes in association with intracellular membranes and the 27.6 kb genome encodes replicase proteins and four major structural proteins [188].

IBV infection can inhibit cell growth by inducing cell cycle arrest in G2 and S-phases in both wild type and p53-null cells [189,190]. Infection of cultured cells with IBV activates the ATR-CHK1 pathway underlined by phosphorylation of H2AX, RPA2 and CHK1 [104]. Furthermore, inhibition of ATR by RNA interference and small molecule inhibitors reduces coronavirus replication while inhibition of DNA-PK and ATM has no effect [104]. DNA replication stress may be induced by IBV via an interaction between the coronavirus non-structural protein nsp13 and DNA polymerase δ which can then lead to ATR activation and S-phase arrest [104]. This ATR-induced cell cycle arrest appears to promote conditions that are favourable to both viral RNA and cellular DNA replication [104,189,190]. Although DNA duplication occurs in the nucleus while coronaviruses replicate exclusively in the cytoplasm, it has been suggested that S-phase extension benefits both processes because IBV can mediate the translocation of abundant host factors between the nucleus and sites of viral replication [104,191].

## 17. Influenza A Virus

Influenza A is an enveloped virus that is part of the *Orthomyxoviridae* family that also contains type B and C influenza viruses. Influenza A has a negative-sense single-stranded RNA genome consisting of eight ribonucleoprotein complexes that encode 11 viral genes [192]. Following cell entry, the negative-sense viral RNA is transported to the nucleus where it provides a template for production of positive-sense mRNA molecules which are then exported to the cytoplasm for translation of viral proteins [192]. Influenza viruses target cells in the upper respiratory tract and are a significant source of human morbidity and mortality through the development and spread of flu. Several subtypes of influenza A have been identified based on variations in viral envelope surface proteins, haemagglutinin (HA) and neuraminidase (NA) [193].

Comet assays have revealed that the influenza A subtype H3N2 can cause DNA damage in leukocytes as early as 2 h post infection [106]. DNA damage was maximal after 24 h although significant cell death was not observed until after 96 h suggesting that infected cells may continue to proliferate in the presence of deleterious mutations. A subsequent study found that infection with the same influenza subtype causes extensive chromatin condensation and fragmentation of DNA into oligonucleosomes consistent with apoptotic cell death [194]. Another major influenza A subtype, H1N1, was shown to induce DNA strand breaks, measured by appearance of γH2AX foci, in both epithelial cells and immune cells [105]. DNA damage persisted after viral clearance and correlated with elevated oxidative stress associated with viral infection. The host inflammatory response appears to be at least partially responsible for DNA damage caused by influenza A infection which subsequently plays a role in localised tissue damage that characterises viral disease. It has yet to be determined which specific influenza A proteins are responsible for DNA damage induction and whether particular repair pathways are compromised by the virus.

## 18. Conclusions

Viruses with RNA genomes are responsible for the emergence of numerous high-profile infectious diseases that include AIDS and SARS, as well as being the causative agents of several aggressive malignancies. Substantial genetic diversity and high mutation rates in these cases have often confounded therapeutic intervention strategies and intensified the need for greater understanding of RNA virus lifecycles. In this regard, there is considerable interest in how the interaction between these pathogens and essential cellular processes, such as the DDR, contribute to viral propagation and pathogenesis.

While all the viruses mentioned above have the potential to introduce genetic instability during the course of infection, the exact mechanism by which DNA lesions are inflicted is often difficult to establish. Viruses typically modulate cell cycle progression and apoptotic pathways in order to ensure efficient and complete replication of their genetic material. Changes that lead to enhanced cellular proliferation and weakening of cell cycle checkpoints can result in replication-induced DNA damage that fails to trigger cell cycle arrest or activation of DNA repair pathways. Furthermore, increased generation of ROS is a common feature of RNA virus infection and is a well-characterised source of endogenous DNA damage [183,195]. The SSBs and base damage typically inflicted by ROS can frequently lead to more deleterious DSBs by impeding DNA replication machinery resulting in fork stalling and collapse [196]. While the process of retroviral integration triggers upregulation of DNA repair pathways, several retroviral proteins are known to cause DNA damage and suppress DNA repair. As such, RNA viruses may have conflicting interactions with the DDR at several stages during their replicative cycles and can potentially inflict DNA damage through both direct and indirect mechanisms.

While DDR activation following viral entry into the cell can represent an antiviral response that leads to apoptosis of the infected cell, RNA viruses can also selectively trigger DDR signalling to promote cellular conditions that are favourable for viral replication, as is the case with many DNA viruses. The Rift Valley fever virus (RVFV), a negative-strand RNA virus of the *Bunyaviridae* family, encodes a non-structural (NSs) protein that causes S-phase arrest by activating the ATM-CHK2 pathway [110]. Chemical inhibition of ATM signalling during RVFV infection both rescues cells from S-phase stasis and significantly reduces levels of viral replication. In this case, it appears that the link between DDR activation and cell cycle regulation is exploited to ensure maximal production of progeny virus. The NSs protein of another member of the *Bunyaviridae* family, the La Crosse virus (LACV), inhibits the type 1 interferon response through degradation of the RNA polymerase II subunit RPB1 [109]. There is evidence that selective activation of DDR components by NSs could be crucial to promoting RPB1 proteasomal degradation. In the case of LACV, limited stimulation of DDR pathways may be part of a strategy to moderate the innate immune response while detrimental effects of wider DDR activation are circumvented. RNA viruses can also acquire a survival advantage by targeting specific DDR proteins. The HIV-1 Tat protein, for example, can suppress the activity of Tip60, a histone acetyltransferase involved in ATM activation. This interaction leads to the suppression of the apoptotic response following DNA damage and could therefore promote viral replication through prolonged survival of the host cell [197]. HIV-1 Tat also downregulates expression of DNA-PKcs, a core factor in NHEJ repair, which sensitises Tat-expressing cells to the effects of IR [198]. More recently this interaction has been shown to impair the role of DNA-PKcs in B-cell antibody class switch recombination, providing a possible mechanism in which HIV-1 could regulate humoral immunity [199].

Regardless of the exact source of DNA damage or consequence of DDR activation during RNA virus lifecycles, increased genetic instability plays a considerable role in the pathogeneses of the viruses discussed here and is likely to be a key factor driving cellular transformation by those species associated with tumour development. Currently, specific cases regarding interactions between RNA viruses and the DDR are less numerous than in DNA viruses and understandably research has focused on those species that are important human health concerns. However, there is no doubt that more examples and increasing layers of complexity will emerge as the field develops.
